# Expression of Circulating MicroRNAs Linked to Bone Metabolism in Chronic Kidney Disease-Mineral and Bone Disorder

**DOI:** 10.3390/biomedicines8120601

**Published:** 2020-12-12

**Authors:** Maria P. Yavropoulou, Vasilios Vaios, Polyzois Makras, Panagiotis Georgianos, Anastasios Batas, Dimitrios Tsalikakis, Alexandros Tzallas, Georgios Ntritsos, Stefanos Roumeliotis, Theodoros Eleftheriadis, Vassilios Liakopoulos

**Affiliations:** 1 Endocrinology Unit, The First Department of Propaedeutic and Internal Medicine, Medical School, National and Kapodistrian University of Athens, GR 11527 Athens, Greece; 2 Department of Medical Research, 251 Hellenic Air Force & VA General Hospital, GR 11525 Athens, Greece; pmakras@gmail.com (P.M.); a.batas@outlook.com (A.B.); 3 Division of Nephrology and Hypertension, The First Department of Internal Medicine, AHEPA Hospital, School of Medicine, Aristotle University of Thessaloniki, GR 54636 Thessaloniki, Greece; vvaios_85@yahoo.gr (V.V.); pangeorgi@yahoo.gr (P.G.); st_roumeliotis@hotmail.com (S.R.); liakopul@otenet.gr (V.L.); 4 Department of Electrical & Computer Engineering, University of Western Macedonia, GR 50100 Kozani, Greece; dtsalikakis@uowm.gr; 5 Department of Informatics & Telecommunications, School of Informatics & Telecommunications, University of Ioannina, GR 47100 Arta, Greece; tzallas@uoi.gr (A.T.); gntritsos@uoi.gr (G.N.); 6 Department of Nephrology, Medical School, University of Thessaly, GR 41110 Larissa, Greece; teleftheriadis@yahoo.com

**Keywords:** CKD-MBD, circulating microRNAs, end stage renal disease, trabecular bone score, osteoporosis

## Abstract

The pathophysiology of chronic kidney disease–mineral and bone disorder (CKD-MBD) is complex and multifactorial. Recent studies have identified a link between microRNAs (miRNAs) and bone loss. In this study, we investigated the expression of miRNAs in CKD-MBD. In this case-control study, we included thirty patients with CKD-MBD (cases) and 30 age- and gender-matched healthy individuals (controls). Bone mineral density (BMD) and trabecular bone score (TBS) evaluation was performed with dual X-ray absorptiometry. The selected panel of miRNAs included: hsa-miRNA-21-5p; hsa-miRNA-23a-3p; hsa-miRNA-24-2-5p; hsa-miRNA-26a-5p; hsa-miRNA-29a-3; hsa-miRNA-124-3p; hsa-miRNA-2861. The majority of cases had low BMD values. The relative expression of miRNA-21-5p was 15 times lower [fold regulation (FR): −14.7 ± 8.1, *p* = 0.034), miRNA-124-3p, 6 times lower (FR: −5.9 ± 4, *p* = 0.005), and miRNA-23a-3p, 4 times lower (FR: −3.8 ± 2.0, *p* = 0.036) in cases compared to controls. MiRNA-23a-3p was significantly and inversely correlated with TBS, adjusted for calcium metabolism and BMD values (beta = −0.221, *p* = 0.003, 95% CI −0.360, −0,081) in cases. In a receiver operating characteristic (ROC) analysis, expression of miRNA-124-3p demonstrated 78% sensitivity and 83% specificity in identifying CKD patents with osteoporosis. Serum expression of miRNAs related to osteoblasts (miRNA-23a-3p) and osteoclasts (miRNA-21-5p, miRNA-124-3p) is significantly altered in patients with CKD-MBD.

## 1. Introduction

Chronic kidney disease–mineral and bone disorder (CKD-MBD) is a systemic disorder that presents with low bone mass and impaired calcium metabolism along with vascular and/or soft tissue calcifications in patients with chronic kidney disease (CKD) [1]. It is associated with increased risk of fragility fractures, cardiovascular disease, and mortality pointing to the systematic complications of the CKD-induced impaired calcium and phosphate metabolism [2]. In particular, patients on hemodialysis (HD) present with a four times higher relative risk of hip fracture [3], more than 50% prevalence of a vertebral fracture [4], and up to 10 to 20 times higher cardiovascular mortality compared to the general population [5]. In addition, bone loss and fracture risk remain serious complications after kidney transplantation, associated with high morbidity and mortality, and high economic costs [6].

Although the Kidney Disease Improving Global Outcomes (KDIGO) guidelines suggest bone mineral density (BMD) measurements with dual energy X-ray absorptiometry (DXA) for the assessment of fracture risk in CKD-MBD patients [2], BMD is not as accurate as in the general population and it tends to underestimate the risk of fragility fractures [7,8,9]. DXA measurements may also offer the assessment of trabecular bone score (TBS), which is an additional diagnostic tool that provides important information about the status of bone microarchitecture at the appendicular skeleton [10]. We and others have shown that TBS is significantly reduced in CKD patients on HD independent of BMD values [10,11,12,13]. Both BMD and TBS measurements, however, are static tests that cannot provide information on the dynamic bone turnover status or the molecular mechanisms linked to the pathophysiology of CKD-MBD.

MicroRNAs (miRNAs) are a class of small, non-coding endogenous RNAs (21–25 nucleotides in length), that play an important regulatory role in various cellular processes in mammals by targeting specific mRNAs for degradation or translation repression. Recent advances in molecular genetics have revealed the molecular pathways and the regulatory mechanisms of miRNAs in the pathophysiology of human diseases, while several preclinical and clinical trials are currently ongoing for miRNA-based therapeutics. A number of studies have demonstrated significant alterations in the expression profile of various miRNAs in CKD associated with the progression of CKD of different etiologies, such as diabetic nephropathy, lupus nephritis, focal segmental glomerulosclerosis, and IgA nephropathy [14]. Neal et al. were the first to use RT-qPCR to show that certain circulating miRNAs are decreased in various stages of CKD [15]. Relative expression of miRNA-155 and miRNA-223 was increased in the serum of patients with CKD, but decreased in renal transplant recipients [16], while serum expression of miRNA-125b, miRNA-145, and miRNA-155 was decreased in a cohort of 90 CKD patients at stage 3 and 4 [17].

Data, however, are scarce regarding the role of miRNAs on bone metabolism in CKD-MBD. In our previous studies, we have identified a particular expression profile of miRNAs targeting genes related to both osteoclast and osteoblast function in postmenopausal osteoporosis (PMO) [18,19,20]. In the present analysis, we sought to investigate whether the expression profile of circulating miRNAs that we have previously shown to be differentially expressed in PMO is also impaired in patients with CKD-MBD.

## 2. Patients and Methods

This was a preliminary case-control study with analysis of data in a specific time point. Thirty patients (22 males) with end-stage renal disease (ESRD) undergoing HD that were under regular follow-up at the Dialysis Unit of AHEPA University Hospital (cases) were matched for age, BMI, and gender with healthy individuals with normal renal function (GFR > 60mL/min) (controls), recruited by AHEPA Hospital’s personnel in a ratio 1:1. Exclusion criteria were diabetes mellitus; impaired liver function; malignancy; diseases known to affect bone turnover other than osteoporosis, such as Paget’s disease of bone; primary hyperparathyroidism; hypo- or hyperthyroidism; medication that affects bone turnover such as bisphosphonates, calcimimetics, and glucocorticoids at any dosage or form at the last 6 months before enrollment. All participants gave their informed consent. The study was approved by the Scientific Review Board of AHEPA University Hospital (protocol number 51741, approval date 455/26-02-2015) [12]. 

### 2.1. Medical Records 

Clinical data such as patients’ age, gender, BMI, dialysis vintage, blood pressure during dialysis, etiology of CKD, and concomitant medication were retrieved from the hospital’s database in compliance with the hospital’s general data protection regulation (GDPR). The underlying cause of CKD in our patient’s cohort was diabetic nephropathy (*n* = 10), hypertensive nephropathy (*n* = 2), chronic obstructive nephropathy (*n* = 1), amyloidosis (*n* = 1), focal segmental glomerulonephritis (*n* = 1), membranoproliferative glomerulonephritis (*n* = 2), IgA nephropathy (*n* = 1), immunotactoid glomerulopathy (*n* = 1), polycystic kidney disease (*n* = 1), unknown cause (*n* = 10). The proportion of unknown causes of CKD in our cohort (approximately 33%) is in accordance with the Greek CKD population and similar to European or US registries. In the European Renal Association-European Dialysis and Transplant Association (ERA-EDTA) annual report for 2018, unknown etiology for ESRD was accounted for 37% of the incident Greek patients, 30.1% of the prevalent Greek patients, and 27% of the European ESRD patients (https://era-edta-reg.org/files/annualreports/pdf/AnnRep2018).

### 2.2. BMD Measurements 

BMD measurements were performed by DXA (Lunar Prodigy, General Electric, San Francisco, CA, USA) at the lumbar spine (L1–L4; LS-BMD), at 2 femoral sites: femoral neck [FN-BMD] and total femur [TH-BMD]), and at the lower 1/3 of the nondominant radius. The coefficients of variations were between 2.5% and 3.7% at LS, 2.2% and 3.1% at FN, 1.5% and 2.7% at TH, and 1.3% and 2.9% at the 1/3 of the radius, as previously described [12]. Osteoporosis was defined as T-score of <−2.5 SD [21,22]. TBS was evaluated in patients ESRD and controls in the same regions as those used for LS-BMD (L1–L4) using TBS iNsight (Version 1.8, Med-Imaps, Pessac, France). The coefficient of variation for TBS was between 2.2% and 3.5% and it did not vary among the measured vertebrae, as previously reported [12]. All patients on dialysis had standardized radiographs in anteroposterior and left lateral projections of the thoracic spine and the LS. 

### 2.3. Blood Sampling and Isolation of MicroRNAs from the Serum

Following an overnight fast, morning blood samples were obtained from both cases and controls. Serum was separated and stored at −80 °C until further analysis. MiRNAs were extracted from 200 µL of serum samples using the miRNeasy Serum/Plasma Kit, according to the manufacturer’s instruction (Qiagen GmbH, Hilden, Germany) as previously described [18,19,20]. A synthetic RNA sequence (spike ins: C. Elegans miR-39) was added in an appropriate amount to serum preparations after homogenization with the QIAzol lysis reagent to control for variations in recovery and amplification efficiency between RNA preparations. As a carrier, we used 1.25 mg/mL bacteriophage MS2 RNA.

### 2.4. Reverse Transcription and PCR Analysis

Reverse transcription was performed with the miScript II RT Kit (Qiagen GmbH, Hilden, Germany) and 1 µL was used as template for each single miRNA assay, according to the manufacturer’s instruction. SnoRNAs (SNORD95, SNORD96A) and snRNA (RNU6-2) were used to normalize for variability in sample loading and real-time RT-PCR efficiency. Cycling was performed under standardized conditions with QuantiTect^®^ SYBR Green PCR Master Mix (Qiagen GmbH, Hilden, Germany) on the QIAGEN Rotor-Gene Q (Corbett Rotor-Gene 6000) real-time PCR cycler, as previously described [20]. Performance of PCR was done in triplicates. 

#### Quality Control 

All cDNAs used in the analysis expressed the spike-in control at normal levels [cycle thresholds (Ct) < 22 cycles].

### 2.5. MicroRNA Primer Assays

We used the miRNA primer assays that we have previously described to be differentially expressed in patients with PMO compared to controls with normal BMD [18,19,20]. The selection of this specific panel of microRNAs was based on their role on bone metabolism (targeting both osteoblast and osteoclast function) (Table 1). In particular, we searched for changes in the relative expression of miRNA-21-5p, miRNA-23-3p, miRNA-24-2-5p, miRNA-26a-3p, miRNA-29a-3p, miRNA-124-3p, and miRNA-2861. As in our previous studies, additional search in the following databases: 1. miRBase [23], 2. DIANA TOOLS [24], 3. PicTar [25] 4. miRDB [26], 5. TargetScanHuman [27], 6. miRGator [28], and 7. microRNA [29], was performed for the confirmation of the biological targets of the selected miRNAs in humans, searching for 8mer, 7mer, and 6mer sites that match the seed region for each miRNA, using conserved sites and the best cumulative scores (Table 1). 

### 2.6. Biochemical Assays

Corrected total serum calcium (Ca) was calculated as follows: corrected total calcium (mg/dL) = total calcium + 0.8 × (4 − albumin [mg/dL]). 

Serum parathyroid hormone (PTH) levels (pmol/L) were measured by an electrochemiluminescence immunoassay (Cobas; Roche, West Sussex, UK). Minimum detectable concentration is 0.1 pmol/L, and the within-run and total run assay coefficients of variation are between 0.6%–2.8% and 1.6%–3.4%, respectively. 

Serum 25-OH vitamin D levels (nmol/L) were measured using an RIA (RIA; DiaSorin, Sallugia, Italy). Minimum detectable concentration is 3.75 nmol/l, and intra-assay and total coefficient of variation for analytical imprecision of this assay is <12.5% and <4%, respectively. 

Bone markers were measured by a second-generation electrochemiluminescence immunoassay on a Cobas e411 automated analyzer (Roche Diagnostics, Mannheim, Germany) as previously described [18]. The measurement range and total analytical imprecisions are: for C-terminal cross-linking telopeptide of type I collagen (β-CTX), 10 to 6000 ng/L and 3.5%, respectively, and for total procollagen type 1 N-terminal propeptide (P1NP), 5 to 1200 ng/mL and 4.5%, respectively.

### 2.7. Statistical Analysis 

All statistical analyses were performed with SPSS Statistics 25.0 (SPSS Inc., Chicago, IL, USA). We used Shapiro–Wilk’s test to assess for normality of distributions and we present our results as mean ± SD or mean (range), as applicable. 

Student’s t-test was used for mean comparison of parametric variables and Mann–Whitney U-test if otherwise distributed. Pearson’s or Spearman’s test were used for parametric and nonparametric correlations, respectively. 

In the group of patients with CKD on HD (cases), linear regression analysis was performed to examine the association between the relative serum expression of the tested miRNAs on the BMD, adjusted for Ca, phosphate (PO4), alkaline phosphatase (ALP), PTH, and 25-OH vitamin D. Receiver-operating characteristic (ROC) curves were analyzed to assess the specificity and sensitivity of the relative expression of specific miRNAs in identifying osteoporotic patients within our cohort of CKD patients. All *p*-values are two-sided and a value of *p* < 0.05 was considered statistically significant.

We used QIAGEN Website for analysis of the PCR data supported by SaBiosciences (miRNA primer assay data analysis version 3.5, GeneGlobe Data Analysis). All analyses were based on fold-change (2^(−Delta Delta Ct)), defined as the normalized gene expression (2^(−Delta Ct)) in cases divided by the normalized gene expression (2^(−Delta Ct)) in controls. Fold-change values less than one were defined as down-regulation of the gene expression, and fold-change values greater than one were defined as up-regulation, accordingly. The *p* values were calculated based on a Student’s t-test of the replicate 2^(−Delta Ct) values for each gene in cases and controls. Mean Ct values less than 33 were used as the cutoff threshold. 

## 3. Results

### 3.1. Study Population

Demographic, anthropometric, and biochemical characteristics of the study cohort are presented in Table 2. 

None of the participants had a history of a prior vertebral or non-vertebral fracture. Serum 25-OH vitamin D levels were significantly lower in cases compared to controls with 33% of cases suffering from severe vitamin D deficiency (defined as 25-OH vitamin D levels < 25 nmol/l), while 100% of the cases had secondary hyperparathyroidism, defined as PTH levels > 7 pmol/L according to the reference values given by the hospital’s central laboratory. Bone mass was also significantly lower at all skeletal sites tested and similarly, TBS values were significantly lower in cases compared to controls (Table 2). According to WHO criteria for BMD measurements, 27 (90%) of the cases had low bone mass, of whom 16 (53%) were osteoporotic and 11 (36%) osteopenic. On the contrary, only five (16%) of the controls were osteoporotic with the rest being within the normal range of BMD. Serum levels of bone turnover markers were marginally but significantly higher in cases compared to controls (Table 2). 

### 3.2. Differential Expression of the Selected Panel of MiRNAs Linked to Bone Metabolism in the Serum of Patients with CKD on HD Compared to Controls

**Figure 1:**
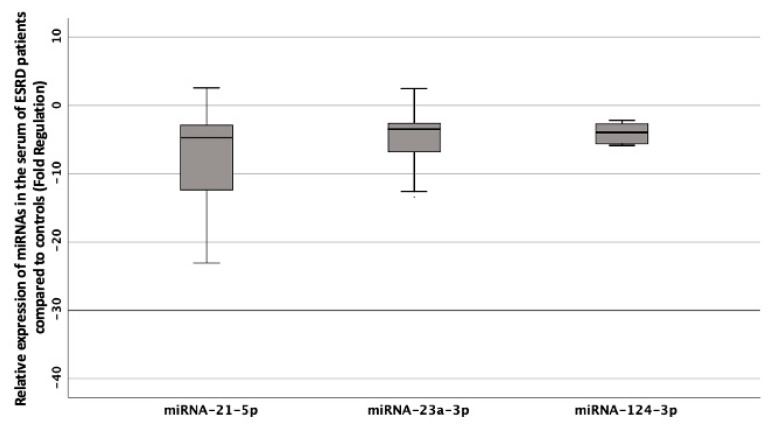
Box plot of the relative expression of the circulating miRNA-21-5p, miRNA-23a-3p, and miRNA-124-3p in cases compared to controls. Values are expressed as gene fold regulation and are displayed in a standardized way (*n* = 30; 25th percentile; median; 75th percentile,). The error bars are showing the minimun and the maximum values. miRNA, microRNA

Three out of the seven tested miRNAs were significantly down-regulated in cases compared to controls (Table 3). In particular, the relative serum expression of miRNA-21-5p was approximately 15 times lower (fold regulation −14.7 ± 8.1, *p* = 0.034), miRNA-124-3p, 6 times lower (fold regulation −5.9 ± 4, *p* = 0.005), and miRNA-23a-3p, 4 times lower (fold regulation −3.8 ± 2.0, *p* = 0.036) in cases compared to controls (Figure 1).

Within the group of cases, miRNA-23a-3p was significantly and inversely correlated with TBS (rho = −0.503, *p* = 0.005), and this correlation remained robust when adjusted for the parameters of Ca metabolism and BMD values (beta = −0.221, *p* = 0.003, 95% CI −0.360, −0,081).

**Figure 2:**
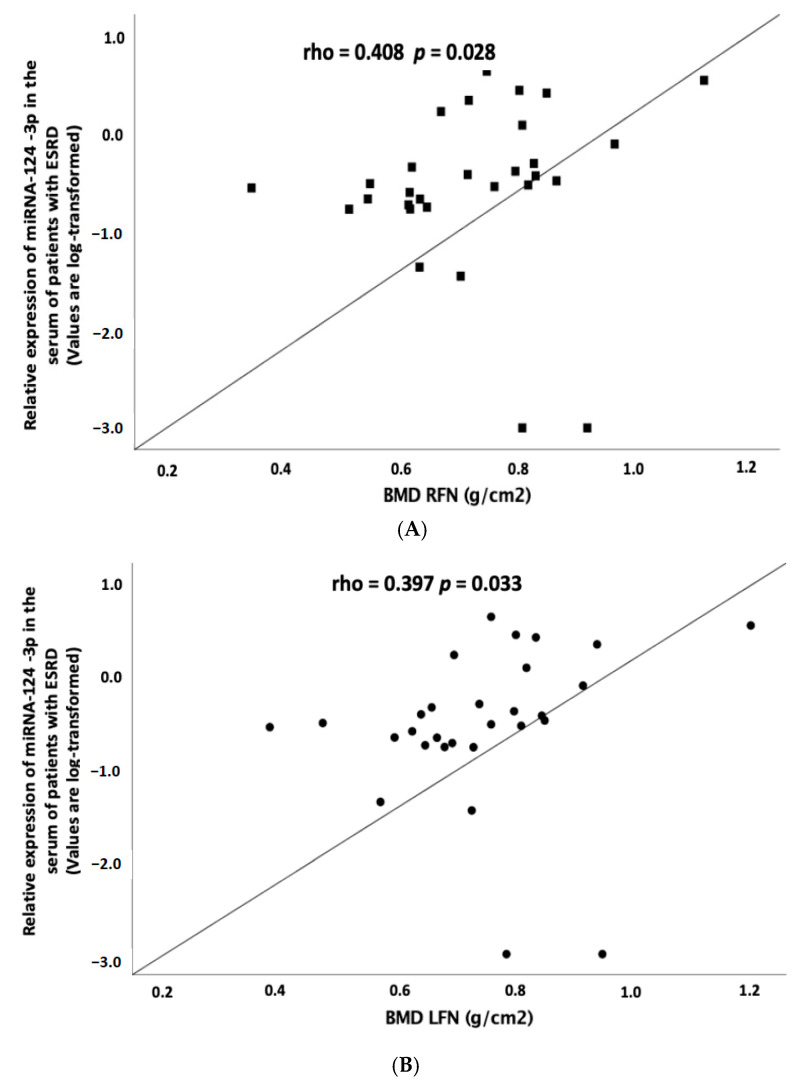
Correlation of the relative expression of miRNA-124-3p in the serum of ESRD patients on hemodialysis (HD) (cases) with (**A**) BMD in RFN and (**B**) BMD in LFN. The relative expression of miRNA-124-3p is log transformed. BMD, bone mineral density; LFN, left femoral neck; RFN, right femoral neck; ESRD, end-stage renal disease; miRNA, microRNA

BMD values at right femoral neck (RFN) and left femoral neck (LFN) but not LS were also significantly correlated with the relative expression of miR-124-3p (Figure 2A,B), but these associations did not remain significant after adjusting with the parameters of calcium metabolism.

No correlations were found between the relative serum expression of the tested miRNAs and the serum values of the calcium metabolism parameters, the bone turnover markers, or the years of dialysis.

A ROC analysis was performed for the differentially expressed miRNAs in the serum of ESRD patients on HD to assess their potential value in distinguishing between cases with and without osteoporosis.

**Figure 3:**
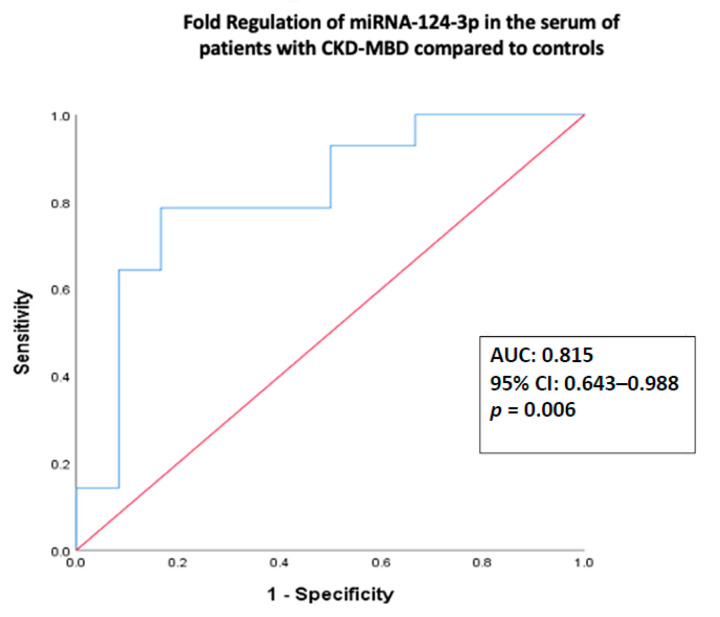
Area under the curve (AUC) of receiver-operating characteristic (ROC) for the relative expression of miRNA-124-3p and ROC curve. AUC, 95% confidence interval (95% CI), and nominal *p* values are demonstrated. The relative expression of miRNA-124-3p is log-transformed.

The associated area under the curve (AUC) of the relative serum expression of miRNA-124-3p on cases compared to controls was 0.815 (95% CI 0.643–0.988, *p* = 0.006) (Figure 3), showing a sensitivity of 78% and specificity of 83% in identifying ESRD patients with low BMD in the osteoporotic range (T-score < −2.5 at FN and/or LS). The respective AUC for miRNA-21-5p was 0.488 (95% CI 0.234–0.742, *p* = 0.13) and for miRNA-23a-3p was 0.607 (95% CI 0.339–0.875, *p* = 0.447).

## 4. Discussion

In the present study, we investigated the relative expression of circulating miRNAs linked to bone metabolism, in our group of patients with ESRD on HD. Bone loss and impaired calcium metabolism in this group of patients is associated with systematic disorders of the vascular axis and thus the identification of the implicated underlying molecular mechanisms is of critical importance towards a prompt diagnosis and an optimal management of CKD-MBD. We have shown here an altered expression pattern of circulating miRNAs that target function and differentiation of both osteoblasts (miRNA 23a-3p) and osteoclasts (miRNA-21-5p and miRNA-124-3p) in our patients. In particular, we report significant down-regulation of the relative serum expression of miRNA-21-5p, miRNA-23a-3p, and miRNA-124-3p in our patients with CKD-MBD compared to age and gender matched controls with normal renal function. 

MiRNA-21-5p and miRNA-124-3p regulate osteoclastogenesis through opposite directions. MiRNA-21-5p is highly expressed in osteoclast precursors and is up-regulated during receptor activator of nuclear factor kappa-B ligand (RANKL)-induced osteoclastogenesis [30,31]. In addition, mice models lacking miRNA-21-5p display inhibition of bone resorption and osteoclast function [32]. On the other hand, expression of miRNA-124 inhibits osteoclastogenesis by suppressing nuclear factor of activated T-cells (NFATc1) and RANKL-mediated osteoclast differentiation of mouse bone marrow macrophages [38]. Thus, decreased relative expression of both miRNA-124-3p and miRNA-21-5p regulate osteoclastogenesis in a fine-tuned mechanism mainly through interfering with the RANKL pathway. RANKL seems to play a critical role in the regulation of bone metabolism in CKD-MBD and is considered a significant link between vascular calcification [40], immune dyregulation [41], and bone disease in these patients. The alterations in the expression of miRNAs that interfere with the RANKL signaling lends further support to the key role of this cytokine in the pathogenesis of CKD-MBD. 

Expression of miRNA-21 and miRNA-124 has been previously reported in different conditions of CKD both in animal models and in humans.

In particular, in CKD mice models miRNA-21 contributes to epithelial disease in response to injury and development of fibrosis, while miRNA-21−/− mice are protected against the development of kidney fibrosis [42]. In humans, circulating and urinary expression of miRNA-21 levels are significantly increased in patients with severe interstitial fibrosis [43].

Expression of miRNA-124 was also found significantly reduced in patients with active lupus nephritis (LN) compared with patients with non-active LN and was negatively correlated with the mRNA expression in the serum of interleukins (IL) 1β and 6, tumor necrosis factor-alpha (TNF-α), and TNF receptor associated factor-6 (TRAF6) [44].

Chronic inflammation due to chronic exposure to uremic toxins, increased intracellular oxidative stress, and endothelial dysfunction has a significantly negative impact on bone turnover and cardiovascular system of ESRD patients [45,46]. A potential role of deregulated miRNAs in this link deserves further investigation.

MiRNA-23a-3p targets osteoblastogenesis and osteoblast function through inhibition of the *RUNX2* mRNA translation in terminally differentiated osteoblasts [33]. In vivo studies have demonstrated that down-regulation of miRNA-23a-3p expression increases osteoblasts proliferation and differentiation through unleashing its negative effect on *RUNX2* expression and WNT/β-catenin signaling in osteoporotic rats [34]. Serum expression of miRNA-23a-3p has been studied in patients that developed acute kidney injury (AKI) after an acute myocardial infarction (AMI), showing significant down-regulation in this group of patients compared to those that did not develop AKI [47].

In the present study, significant down-regulation of both miRNA-23a-3p and miRNA-124-3p is expected to increase osteoblastogenesis and osteoclastogenesis, and this is in line with the high bone turnover status of our patients, defined by the high PTH levels and the values of the bone turnover markers P1NP and β-CTX. On the other hand, significant down-regulation of miRNA-21-5p would decrease osteoclastogenesis, probably as a regulatory feedback mechanism of bone tissue to attenuate enhancement of osteoclastogenesis caused by secondary hyperparathyroidism and high bone turnover status in CKD-MBD.

In addition, we found significant correlation of the relative expression of miRNA-23a-3p with bone microarchitecture, as assessed by the TBS, after adjustment for ΒΜD values and parameters of calcium metabolism that are significantly altered in CKD-MBD.

Lower TBS values in ESRD patients are associated not only with increased prevalent or incident fragility fractures [48,49], but also with increased prevalence of cardiovascular events and increased risk of mortality [50]. Therefore, correlation of the low TBS values with the down-regulation of miRNA-23a-3p expression independent of the BMD and the impaired calcium metabolism in our cohort of ESRD patients lends further support to the epigenetic role of altered miRNAs in the pathophysiology of CKD-MBD.

Our results showing altered expression of circulating miRNA-21a-5p, miRNA-23a-3p, and miRNA-124-3p in patients with CKD-MBD compared to controls with normal renal function is in line with our previous results in women with PMO [20], and in patients with acquired immune deficiency syndrome (AIDS) and low bone mass [51], suggesting a common pattern of miRNA expression profile in bone loss independent of the underlying cause. In addition, in the present study, the relative expression of miRNA-124-3p showed 78% sensitivity and 83% specificity in identifying patients with CKD-MBD and osteoporosis based on DXA measurements, suggesting its role as a novel diagnostic biomarker and potential therapeutic target in this patient group.

Our study has several limitations. First, its cross-sectional design did not allow us to have a better view of changes in the bone-remodeling status over time in this group of patients that are characterized by a significantly dynamic and complex bone and calcium metabolism. Second, the relatively small number of participants and the pre-specified profile of the miRNAs investigated in the serum. Third, one could argue that secondary hyperparathyroidism might probably be the cause of the miRNAs’ altered expression in our patients. However, the common pattern of miRNA changes in CKD-BMD, PMO, and AIDS-induced bone disease suggests that these miRNAs are probably significant key factors in the pathophysiology of bone loss in all osteoporotic cases.

Nevertheless, this is the first study investigating the molecular profile of circulating miRNAs linked to bone metabolism and osteoporosis in patients with CKD-MBD and suggests an epigenetic molecular component in this group of patients and deregulation of both osteoclastogenesis and osteoblastogenesis at the tissue level.

Novel biomarkers are urgently needed for the diagnosis and monitor of CKD-MBD progression in order to promptly identify specific CKD-MBD-associated complications and guide appropriate management. MiRNAs are suitable candidates for noninvasive biomarkers, as they can be tissue- and cell-specific, are very stable in the blood, and their serum expression reflects the pathophysiology of the diseased tissues.

## Figures and Tables

**Table 1:** Pre-specified panel of selected microRNAs linked to bone metabolism.

MiScript Primer Assay	Gene Symbol	Predicted Target-Genes	MicroRNA Sequence	Predicted Mechanism of Action
MS00009079	hsa-miRNA-21-5p	*SPRY1; PDCD4; FASLG*	5′UAGCUUAUCAGACUGAUGUUGA	Increases osteoclastogenesis and is up-regulated during RANKL-induced osteoclastogenesis [30,31,32].
MS00031633	hsa-miRNA-23a-3p	*RUNX2; SATB2*	5′AUCACAUUGCCAGGGAUUUCC	Decreases osteoblastogenesis, through inhibition of *RUNX2* translation [33,34].
MS00009205	hsa-miRNA-24-2-5p	*TCF-1; CALB1; SATB2*	5′UGCCUACUGAGCUGAAACACAG	Decreases osteogenic differentiation through targeting the expression of transcription factor *TCF-1* in osteoblastic cells [35].
MS00029239	hsa-miRNA-26a-5p	*ΤΟΒ1; IGF-1*	5′UUCAAGUAAUCCAGGAUAGGCU	Increases bone formation through repressing TOB1 protein expression, negative regulator of BMP/Smad signaling pathway [36].
MS00003262	hsa-miRNA-29a-3p	*SPARC*	5′UAGCACCAUCUGAAAUCGGUUA	Decreases osteonectin-bone matrix protein-synthesis [37].
MS00006622	hsa-miRNA-124-3p	*NFATC1; NFATC2*	5′UAAGGCACGCGGUGAAUGCC	Decreases osteoclastogenesis by suppressing NFATc1 [38].
MS00042140	hsa-miRNA-2861	*HDAC5*	5′GGGGCCUGGCGGUGGGCGG	Increases osteoblastogenesis through repression of *HDAC5* that degrades expression of the *RUNX2 gene* [39].
MS00019789	Cel-miRNA-39-3p	Spike in control	5′UCACCGGGUGUAAAUCAGCUUG	

All selected primers are mature microRNAs. hsa, *homo sapiens*; miRNA, microRNA; *SPRY1*, sprouty homolog 1, antagonist of FGF signaling; *PDCD4*, Programmed cell death protein 4; *FASLG*, tumor necrosis factor ligand; *RUNX2*, Runt-related transcription factor; *SATB2*, SATB homeobox 2; *TCF-1*, T-cell factor-1; *CALB1*, calbindin 1, 28kDa; *TOB1*, Transducer of Erb-2, 1; *IGF-1*, insulin-like growth factor 1; *SPARC*, Secreted protein, acidic, cysteine-rich (osteonectin); *NFATC1*, Nuclear factor of activated T-cells, cytoplasmic, 1; *NFATC2*, nuclear factor of activated T-cells, cytoplasmic, 2; *HDAC5*, Histone deacetyl transferase 5; Cel, Caenorhabditis elegans.

**Table 2:** Anthropometric characteristics and biochemical values of the study group.

Parameters	Patients with CKD on HD (Cases = 30)	Healthy Individuals (Controls, *n* = 30)	*p*-Value *
Age (years)	58.3 ± 12.4	54.7 ± 5.5	0.256
BMI (kg/m^2^)	25.8 ± 3.9	25.5 ± 1.5	0.821
Males, n (%)	22 (73%)	22 (73%)	0.132
Duration of Hemodialysis (years)	5.9 ± 4.7	NA	NA
Smoking, n (%)	4 (13%)	6 (20%)	0.156
LS T-score	−1.7 [(−3.7), (+1.7)]	0.4 [(−1.9), (+0.7)]	0.001
LS-BMD (gr/cm^2^)	0.967 ± 0.21	1.177 ± 0.13	<0.001
TBS	1.144 ± 0.16	1.219 ± 0.15	0.004
LFN T-score	−2.4± 1.1	−0.87 ± 0.93	<0.001
LFN BMD (gr/cm^2^)	0.740 ± 0.15	0.956 ± 0.12	<0.001
LH T-score	−2.2 ± 1.18	−0.6 ± 0.81	<0.001
LH BMD (gr/cm^2^)	0.775 ± 0.17	1.013 ± 0.11	<0.001
RFN T-score	−2.47 ± 1.15	−1.0 ± 0.93	<0.001
RFN BMD (gr/cm^2^)	0.729 ± 0.15	0.940 ± 0.12	<0.001
RH T-score	−2.2 ± 1.15	−0.68 ± 0.78	<0.001
RH BMD (gr/cm^2^)	0.770 ± 0.16	1.003 ± 0.11	<0.001
Radius T-score	−2.83 ± 2.25	−0.38 ± 0.93	<0.001
Radius BMD (gr/cm^2^)	0.366 ± 0.11	0.96 ± 0.09	<0.001
TBS	1.242 ± 0.12	1.280 ± 0.12	0.098
Serum Calcium # (NR: 8.2–10.6 mg/dl)	8.8 ± 0.5	9.2 ± 0.2	0.004
Serum Phosphate (NR: 2.7–4.5 mg/dl)	4.5 ± 1.6	3.2 ± 0.4	0.002
Intact PTH (NR: 1.58–6.03 pmol/l)	32.25 (4.9, 101)	5 (3.7, 6.0)	<0.001
Serum 25-OH-Vitamin D (nmol/l)	31.75 (10.0, 99.2)	55.0 (22.2, 100)	0.040
Serum ALP (U/lt)	122.0 (52, 386)	71 (45, 117)	0.0013
Serum P1NP (ng/mL)	53.8 ± 12.4	46.9 ± 11.9	0.040
Serum β-CTX (ng/l)	420 ± 120.2	330 ± 95.8	0.048
Clinical/morphometric Vertebral Fractures	0	0	NA
Other Fractures	0	0	NA

Values are expressed as mean ± SD for normally distributed variables, or median (range; min, max) for variables without normal distribution, and n, % for categorical variables. * comparisons are performed between cases and controls. #: Ca values are corrected for albumin. NA, not applicable, BMI, body mass index; LS, lumbar spine; BMD, bone mineral density; TBS, trabecular bone score; LFN, left femoral neck; LH, left total hip; RFN, right femoral neck; RH, right total hip; NR, normal range.

**Table 3:** Differential expression pattern of microRNAs linked to bone metabolism in the serum of end-stage renal disease (ESRD) patients compared controls.

MicroRNAs	Fold Regulation	95% CI	*p*-Values
hsa-miRNA-21-5p	−14.7	(−18.8, −3.9)	*p* = 0.034
hsa-miRNA-23a-3p	−3.8	(−8.7, −2.0)	*p* = 0.005
hsa-miRNA-24-2-5p	1.14	(0.80, 2.11)	*p* = 0.23
hsa-miRNA-26a-5p	1.02	(0.05, 4.12)	*p* = 0.32
hsa-miRNA-29a-3p	1.31	(0.01, 2.76)	*p* = 0.36
hsa-miRNA-124	−5.9	(0.97, 9.50)	*p* = 0.005
hsa-miRNA-2861	1.0	(0.02, 3.79)	*p* = 0.51

Comparisons are performed between patients with ESRD and controls. hsa, *homo sapiens*; miRNA, microRNA; ESRD, end-stage renal disease; 95% CI, confidence interval.

## Abbreviations

miRNA	microRNAs
CKD-MBD	Chronic kidney disease-mineral and bone disorder
HD	Hemodialysis
KDIGO	Kidney Disease Improving Global Outcomes
DXA	Dual energy X-ray absorptiometry
TBS	Trabecular bone score
PMO	Postmenopausal osteoporosis
ESRD	End Stage Renal Disease
BMI	Body Mass Index
GDPR	General data protection regulation
BMD	Bone mineral density
LS	Lumbar spine
FN	Femoral neck
TH	Total hip
PTH	Parathyroid hormone
CVA	coefficient of variation for analytical imprecision
Β-CTX	C-terminal cross-linking telopeptide of type I collagen
P1NP	Procollagen type 1 N-terminal propeptide
SD	Standard deviation
RANKL	Receptor activator of nuclear factor kappa-B ligand
NFATc1	Nuclear factor of activated T-cells
LN	Lupus nephritis
IL	Interleukins
TNF-α	Tumor necrosis factor-alpha
TRAF6	TNF receptor associated factor-6
RUNX2	Runt
AKI	Acute kidney injury
AMI	Acute myocardial infarction
TGF-β	Transforming growth factor beta
AIDS	Acquired immune deficiency syndrome

## References

[1] Kidney Disease: Improving Global Outcomes (KDIGO) CKD-MBD Work Group (2009). KDIGO clinical practice guideline for the diagnosis, evaluation, prevention, and treatment of Chronic Kidney Disease-Mineral and Bone Disorder (CKD-MBD). Kidney Int. Suppl..

[2] Ketteler M., Block G.A., Evenepoel P., Fukagawa M., Herzog C.A., McCann L., Moe S.M., Shroff R., Tonelli M.A., Toussaint N.D. (2017). Executive summary of the 2017 KDIGO Chronic Kidney Disease-Mineral and Bone Disorder (CKD-MBD) Guideline Update: What’s changed and why it matters. Kidney Int..

[3] Alem A.M., Sherrard D.J., Gillen D.L., Weiss N.S., Beresford S.A., Heckbert S.R., Wong C., Stehman-Breen C. (2000). Increased risk of hip fracture among patients with end-stage renal disease. Kidney Int..

[4] Fusaro M., Tripepi G., Noale M., Vajente N., Plebani M., Zaninotto M., Guglielmi G., Miotto D., Dalle Carbonare L., D’Angelo A. (2013). High prevalence of vertebral fractures assessed by quantitative morphometry in hemodialysis patients, strongly associated with vascular calcifications. Calcif. Tissue Int..

[5] Coresh J., Longenecker J.C., Miller E.R., Young H.J., Klag M.J. (1998). Epidemiology of cardiovascular risk factors in chronic renal disease. J. Am. Soc. Nephrol..

[6] Dounousi E., Leivaditis K., Eleftheriadis T., Liakopoulos V. (2015). Osteoporosis after renal transplantation. Int. Urol. Nephrol..

[7] Jamal S.A., Chase C., Goh Y.I., Richardson R., Hawker G.A. (2002). Bone density and heel ultrasound testing do not identify patients with dialysis-dependent renal failure who have had fractures. Am. J. Kidney Dis..

[8] Negri A.L., Barone R., Quiroga M.A., Bravo M., Marino A., Fradinger E., Bogado C.E., Zanchetta J.R. (2004). Bone mineral density: Serum markers of bone turnover and their relationships in peritoneal dialysis. Perit. Dial. Int..

[9] Pimentel A., Urena-Torres P., Zillikens M.C., Bover J., Cohen-Solal M. (2017). Fractures in patients with CKD-diagnosis, treatment, and prevention: A review by members of the European Calcified Tissue Society and the European Renal Association of Nephrology Dialysis and Transplantation. Kidney Int..

[10] Martineau P., Leslie W.D. (2017). Trabecular bone score (TBS): Method and applications. Bone.

[11] Dusceac R., Niculescu D.A., Dobre R., Dragne M.C., Tacu C., Peride I., David C., Checherita I., Poiana C. (2018). Chronic hemodialysis is associated with lower trabecular bone score, independent of bone mineral density: A case-control study. Arch. Osteoporos.

[12] Yavropoulou M.P., Vaios V., Pikilidou M., Chryssogonidis I., Sachinidou M., Tournis S., Makris K., Kotsa K., Daniilidis M., Haritanti A. (2017). Bone Quality Assessment as Measured by Trabecular Bone Score in Patients With End-Stage Renal Disease on Dialysis. J. Clin. Densitom..

[13] Yoon H.E., Kim Y., Shin S.J., Hong Y.S., Kang K.Y. (2019). Factors associated with low trabecular bone scores in patients with end-stage kidney disease. J. Bone Miner Metab..

[14] Metzinger-Le Meuth V., Burtey S., Maitrias P., Massy Z.A., Metzinger L. (2017). microRNAs in the pathophysiology of CKD-MBD: Biomarkers and innovative drugs. Biochim. Biophys. Acta Mol. Basis Dis..

[15] Neal C.S., Michael M.Z., Pimlott L.K., Yong T.Y., Li J.Y., Gleadle J.M. (2011). Circulating microRNA expression is reduced in chronic kidney disease. Nephrol. Dial. Transpl..

[16] Brigant B., Metzinger-Le Meuth V., Massy Z.A., McKay N., Liabeuf S., Pelletier M., Sallee M., M’Baya-Moutoula E., Paul P., Drueke T.B. (2017). Serum microRNAs are altered in various stages of chronic kidney disease: A preliminary study. Clin. Kidney J..

[17] Chen N.X., Kiattisunthorn K., O’Neill K.D., Chen X., Moorthi R.N., Gattone V.H., Allen M.R., Moe S.M. (2013). Decreased microRNA is involved in the vascular remodeling abnormalities in chronic kidney disease (CKD). PLoS ONE.

[18] Anastasilakis A.D., Makras P., Pikilidou M., Tournis S., Makris K., Bisbinas I., Tsave O., Yovos J.G., Yavropoulou M.P. (2018). Changes of Circulating MicroRNAs in Response to Treatment With Teriparatide or Denosumab in Postmenopausal Osteoporosis. J. Clin. Endocrinol. Metab..

[19] Yavropoulou M.P., Anastasilakis A.D., Makras P., Papatheodorou A., Rauner M., Hofbauer L.C., Tsourdi E. (2020). Serum Profile of microRNAs Linked to Bone Metabolism During Sequential Treatment for Postmenopausal Osteoporosis. J. Clin. Endocrinol. Metab..

[20] Yavropoulou M.P., Anastasilakis A.D., Makras P., Tsalikakis D.G., Grammatiki M., Yovos J.G. (2017). Expression of microRNAs that regulate bone turnover in the serum of postmenopausal women with low bone mass and vertebral fractures. Eur. J. Endocrinol..

[21] Kanis J.A. (1994). Assessment of fracture risk and its application to screening for postmenopausal osteoporosis: Synopsis of a WHO report. Osteoporos Int..

[22] Kanis J.A., Melton L.J., Christiansen C., Johnston C.C., Khaltaev N. (1994). The diagnosis of osteoporosis. J. Bone Miner Res..

[23] Kozomara A., Griffiths-Jones S. (2014). miRBase: Annotating high confidence microRNAs using deep sequencing data. Nucleic Acids Res..

[24] Paraskevopoulou M.D., Georgakilas G., Kostoulas N., Vlachos I.S., Vergoulis T., Reczko M., Filippidis C., Dalamagas T., Hatzigeorgiou A.G. (2013). DIANA-microT web server v5.0: Service integration into miRNA functional analysis workflows. Nucleic Acids Res..

[25] Reczko M., Maragkakis M., Alexiou P., Grosse I., Hatzigeorgiou A.G. (2012). Functional microRNA targets in protein coding sequences. Bioinformatics.

[26] Wong N., Wang X. (2015). miRDB: An online resource for microRNA target prediction and functional annotations. Nucleic Acids Res..

[27] Agarwal V., Bell G.W., Nam J.W., Bartel D.P. (2015). Predicting effective microRNA target sites in mammalian mRNAs. Elife.

[28] Cho S., Jang I., Jun Y., Yoon S., Ko M., Kwon Y., Choi I., Chang H., Ryu D., Lee B. (2013). MiRGator v3.0: A microRNA portal for deep sequencing, expression profiling and mRNA targeting. Nucleic Acids Res..

[29] Betel D., Koppal A., Agius P., Sander C., Leslie C. (2010). Comprehensive modeling of microRNA targets predicts functional non-conserved and non-canonical sites. Genome Biol..

[30] Sugatani T., Vacher J., Hruska K.A. (2011). A microRNA expression signature of osteoclastogenesis. Blood.

[31] Wang S., Liu Z., Wang J., Ji X., Yao Z., Wang X. (2020). miR21 promotes osteoclastogenesis through activation of PI3K/Akt signaling by targeting Pten in RAW264.7 cells. Mol. Med. Rep..

[32] Hu C.H., Sui B.D., Du F.Y., Shuai Y., Zheng C.X., Zhao P., Yu X.R., Jin Y. (2017). miR-21 deficiency inhibits osteoclast function and prevents bone loss in mice. Sci. Rep..

[33] Hassan M.Q., Gordon J.A., Beloti M.M., Croce C.M., van Wijnen A.J., Stein J.L., Stein G.S., Lian J.B. (2010). A network connecting Runx2, SATB2, and the miR-23a~27a~24-2 cluster regulates the osteoblast differentiation program. Proc. Natl. Acad. Sci. USA.

[34] Dai Y., Zheng C., Li H. (2019). Inhibition of miR-23a-3p promotes osteoblast proliferation and differentiation. J. Cell Biochem..

[35] Zhao W., Wu C., Dong Y., Ma Y., Jin Y., Ji Y. (2015). MicroRNA-24 Regulates Osteogenic Differentiation via Targeting T-Cell Factor-1. Int. J. Mol. Sci..

[36] Li Y., Fan L., Hu J., Zhang L., Liao L., Liu S., Wu D., Yang P., Shen L., Chen J. (2015). MiR-26a Rescues Bone Regeneration Deficiency of Mesenchymal Stem Cells Derived From Osteoporotic Mice. Mol. Ther..

[37] Kapinas K., Kessler C.B., Delany A.M. (2009). miR-29 suppression of osteonectin in osteoblasts: Regulation during differentiation and by canonical Wnt signaling. J. Cell Biochem..

[38] Lee Y., Kim H.J., Park C.K., Kim Y.G., Lee H.J., Kim J.Y., Kim H.H. (2013). MicroRNA-124 regulates osteoclast differentiation. Bone.

[39] Li H., Xie H., Liu W., Hu R., Huang B., Tan Y.F., Xu K., Sheng Z.F., Zhou H.D., Wu X.P. (2009). A novel microRNA targeting HDAC5 regulates osteoblast differentiation in mice and contributes to primary osteoporosis in humans. J. Clin. Invest..

[40] Znorko B., Oksztulska-Kolanek E., Michalowska M., Kaminski T., Pawlak K. (2017). Does the OPG/RANKL system contribute to the bone-vascular axis in chronic kidney disease? A systematic review. Adv. Med. Sci..

[41] Walsh M.C., Choi Y. (2014). Biology of the RANKL-RANK-OPG System in Immunity, Bone, and Beyond. Front. Immunol..

[42] Gomez I.G., Nakagawa N., Duffield J.S. (2016). MicroRNAs as novel therapeutic targets to treat kidney injury and fibrosis. Am. J. Physiol. Renal. Physiol..

[43] Zununi Vahed S., Omidi Y., Ardalan M., Samadi N. (2017). Dysregulation of urinary miR-21 and miR-200b associated with interstitial fibrosis and tubular atrophy (IFTA) in renal transplant recipients. Clin. Biochem..

[44] Zhang L., Zhang X., Si F. (2019). MicroRNA-124 represents a novel diagnostic marker in human lupus nephritis and plays an inhibitory effect on the growth and inflammation of renal mesangial cells by targeting TRAF6. Int. J. Clin. Exp. Pathol..

[45] Eleftheriadis T., Kartsios C., Antoniadi G., Kazila P., Dimitriadou M., Sotiriadou E., Koltsida M., Golfinopoulos S., Liakopoulos V., Christopoulou-Apostolaki M. (2008). The impact of chronic inflammation on bone turnover in hemodialysis patients. Ren. Fail.

[46] Kaminski T.W., Pawlak K., Karbowska M., Mysliwiec M., Pawlak D. (2017). Indoxyl sulfate-the uremic toxin linking hemostatic system disturbances with the prevalence of cardiovascular disease in patients with chronic kidney disease. BMC Nephrol..

[47] Fan P.C., Chen C.C., Peng C.C., Chang C.H., Yang C.H., Yang C., Chu L.J., Chen Y.C., Yang C.W., Chang Y.S. (2019). A circulating miRNA signature for early diagnosis of acute kidney injury following acute myocardial infarction. J. Transl. Med..

[48] Naylor K.L., Prior J., Garg A.X., Berger C., Langsetmo L., Adachi J.D., Goltzman D., Kovacs C.S., Josse R.G., Leslie W.D. (2016). Trabecular Bone Score and Incident Fragility Fracture Risk in Adults with Reduced Kidney Function. Clin. J. Am. Soc. Nephrol..

[49] Aleksova J., Kurniawan S., Elder G.J. (2018). The trabecular bone score is associated with bone mineral density, markers of bone turnover and prevalent fracture in patients with end stage kidney disease. Osteoporos Int..

[50] Yun H.J., Ryoo S.R., Kim J.E., Choi Y.J., Park I., Shin G.T., Kim H., Jeong J.C. (2020). Trabecular bone score may indicate chronic kidney disease-mineral and bone disorder (CKD-MBD) phenotypes in hemodialysis patients: A prospective observational study. BMC Nephrol..

[51] Yavropoulou M., Kolynou A., Makras P., Skoura L., Nanoudis S., Pikilidou M., Tsave O., Metallidis S., Chatzidimitriou D. (2020). AEP131 Changes in the relative expression of circulating microRNAs linked to bone metabolism in HIV-infected Individuals with low bone mass. Endocr. Abstr..

